# COVID-19 Vaccination Among the Arab Bedouin Population: Lessons Learned From a Minority Population

**DOI:** 10.3389/ijph.2022.1604133

**Published:** 2022-03-22

**Authors:** Naim Abu-Freha, Hadel Alsana, Sabri El-Saied, Zuya Azbarga, Muhammad Aloka, Tarek Goda, Muhammad Abu Tailakh

**Affiliations:** ^1^ Arab Medical Association in the Negev, Beersheba, Israel; ^2^ Soroka Medical Center, Beersheba, Israel; ^3^ The Faculty of Health Sciences, Ben-Gurion University of the Negev, Beersheba, Israel

**Keywords:** COVID-19, vaccination, Arab Bedouin, southern Israel, minority, language, culture, awareness

## Abstract

**Objectives:** We investigated the causes of low COVID-19 vaccination rates among a minority population and highlighted interventions for increasing the vaccination rate.

**Methods:** We reviewed the experience during the mass vaccination campaign period among the Arab Bedouin (AB) in Israel, attempting to determine important causes of low vaccination rates and gathered information from real-life experience and through direct contact with the population during the campaign.

**Results:** Causes for low vaccination rates in the AB are related to the health system infrastructure, crisis management strategies, and population characteristics. Long-standing socioeconomic inequalities, limited resources, and language and culture barriers present special challenges to the task of COVID-19 vaccination campaigns. Key interventions for increasing vaccination rates among minority populations include raising awareness, improving vaccination access, and directly targeting risk-groups. To maximize the effectiveness of these interventions they should be culturally adapted and executed according to the needs of each individual target community.

**Conclusion:** Culturally adapted awareness campaigns, interventions, and improved access to vaccines can be accomplished by cooperation between relevant governing and community bodies to increase COVID-19 vaccination rates among minorities.

## 1 Introduction

The ongoing novel coronavirus (COVID-19) pandemic that was traced to Wuhan, China in December 2019, has afflicted racial and ethnic minorities disproportionately [1]. Over the course of months, the virus had spread to most countries of the world. As of February 2022, there have been over 384,000,000 confirmed cases and 5,700,000 deaths worldwide [2]. The devastating medical, economic, and social impact on people around the world has necessitated the development and distribution of a safe and effective vaccine. By April 2020, at least 30 different candidate vaccines were being researched [3]. The BNT162b2 mRNA COVID-19 vaccine manufactured by Pfizer was the first to be approved [4].

The Arab Bedouin (AB) is a subgroup within the Arab minority in Israel that has unique historical, social, and cultural characteristics. In 2019, the size of the AB population in southern Israel was estimated to be 280,000 [5]. This traditionally nomadic, tribal society has rapidly transitioned to a primarily urban population over the span of a single generation in recent years. Approximately 40% of the population still lives in rural areas under semi-nomadic conditions. Some families dwell in difficult-to-access, makeshift structures for extended periods of time without municipal services, paved roads adequate healthcare; many households lack infrastructure for water and electricity [6, 7]. The high rate of consanguinity, high birth rates, and the rapid adoption of a more Western lifestyle have contributed to the rising incidence of congenital and preventable diseases, such as diabetes mellitus and ischemic heart disease [8, 9]. These unique characteristics make this population sector of special interest.

When the first approved vaccine for COVID-19 was made available in December 2020, Israel began its national vaccination campaign, prioritizing at-risk populations. The plan for mass immunization of Israelis was divided into phases, first targeting people above the age of 65 and health workers. The vaccination rate in the general Arab population was low in the first 8 weeks of the campaign, particularly among the AB in southern Israel. As of February 2021, only about 8% of the AB population (in southern Israel) had been vaccinated in comparison with 25% of the total Arab population, and 45% of the general Israeli population [10].

The aim of this review is to elucidate the causes and challenges of low vaccination rates in minority populations and to present successful interventions for correcting these disparities.

## 2 Methods

We reviewed and summarized the causes for the low rate of vaccination among the AB in southern Israel, according to the real-life experiences in the field during Israel’s mass vaccination campaign. During the campaign, several parties [third sector organizations, local council, health maintenance organizations (HMO’s), the ministry of health and others] were active in executing interventions to improve the vaccination rate among the AB in southern Israel. This review is a summary of lessons learned from the direct contact with the population and summarized possible interventions could be conducted in such outbreak.

## 3 Results

### 3.1 Causes of Low COVID-19 Vaccination Rates

There are several reasons for poor vaccination rates among minorities. They can be divided into several categories related to the health system infrastructure, management of the outbreak, characteristics of the vaccine, and demographic factors:

1) Gaps in health system infrastructure.
2) Long-standing neglect of health promotion in the community.
3) General lack of trust in the government and health system before and during the pandemic.
4) Misinformation, lack of access to accurate and scientific data, and the failure of authorities to present information to its target audience in a culturally competent way.
5) Linguistic barriers.
6) Vaccination hesitancy due to suspicion regarding the vaccine’s efficacy and safety in light of its rapid development and distribution.
7) Suspicion related to the decision-making process from the authorities during the crisis.
8) The belief that natural immunity, by infection with COVID-19 rather than by vaccination, that was prominent particularly among young people.
9) Lack of access to the vaccine to all but prioritized populations at the start of the campaign, as well as limited access in peripheral areas throughout the remainder of the campaign, particularly in villages unrecognized by the government.
10) Socioeconomic inequalities: long-standing socioeconomic inequalities among the general Arab population in Israel (and the AB of southern Israel in particular), have greatly influenced the population’s overall health at baseline. These inequalities were only further magnified during the pandemic. Most of the Arab population in Israel lives in separate, primarily Arab villages and cities. Generation of low socioeconomic status and societal neglect have contributed to poor health systems in Arab communities, which in turn correlate with a higher prevalence of chronic diseases such as diabetes mellitus and morbid obesity [12, 13]. Other inequalities relative to the Jewish population manifest in poor infrastructure, educational systems, higher unemployment rates, and crowded living conditions.

### 3.2 COVID-19 Vaccination of Health Workers From a Minority Population

During the time period of this review, a survey on the COVID-19 vaccination rates was conducted by health workers of the same minority as an example of a specific group indirectly related to the outbreak, in term of one of the groups began the vaccination and as a part of the same population with academic backgrounds and heightened pandemic safety awareness. Of the 377 participants, 55.2% were physicians and 50.1% worked at medical center. There were 267 (71.4%) vaccinated subjects, and 41 (10.8%) had not been vaccinated due to having been previously infected with the virus. Arab Bedouin medical staff members who got the vaccine were younger and higher proportion of males.

## 4 Challenges and Barriers During Outbreak

There are unique challenges that researchers and intervening authorities face when reaching out to minority populations during an infectious disease outbreak or other broad scale crises. For containment, contacting a large number of people within a short time period is paramount to quickly raising awareness and slowing the spread of the disease, but the nature of such crises limits opportunities for communication. As such, most meetings with the subjects of our study were conducted virtually. Unfortunately, the challenges of virtual meetings with minority individuals from rural regions are particularly prominent in the Negev area of Israel, where about 40% of the Bedouin population lives without consistent access to electricity and internet.

Another challenge for the COVID-19 vaccination campaign has been limited resources, spread thin over. In addition, resources for the COVID-19 vaccination campaign are limited because they are also allocated for diagnostic tests, treatments, and patient data collection during the outbreak. Minorities, which suffer from a dearth of resources at baseline, are disproportionately impacted during health crises due to poor healthcare access, lack of health education, and worse health status.

Other challenges stem from language and culture barriers. If the unique linguistic and cultural needs of minorities are not considered when managing a crisis, interventions could have a diminished effect or even cause harm.

Finally, racial and ethnic differences in how populations respond to the outbreak complicate the management of the crisis.

## 5 Interventions

In parallel with the launching of widespread vaccination programs, other important interventions have been conducted as part of the national emergency response. Interventions were conducted and coordinated by the health ministry, HMOs, local councils and leaders, the Home Front Command and third sector organizations. Cooperation and a clear division of labor between these bodies has been central to the success of these interventions.

### 5.1 Awareness

Intensive, culturally competent awareness campaigns were necessary to curb the spread of the virus, particularly when addressing the efficacy, safety, and side effects of the vaccine. Involvement of local physicians, researchers, and leaders in planning and conducting awareness activities has been crucial to the success of the vaccination campaign. Such activities have mainly taken place on social media platforms, large zoom meetings, short videos, billboards, and small group meetings. Vaccine promotion conducted by the vaccine priority committee and other authorities was geared toward whatever population that was the current target of vaccine distribution at the time. Initially, the targets were people over the age of 60 and medical workers. Later, this work branched out to include education providers and students in grades [11, 12]. Finally, campaigns adjusted to include any individual over 16 years old that became eligible for the vaccine.

Despite these efforts, the spread of misinformation was influential enough to negatively impact the vaccination rate.

### 5.2 Improved Access to Vaccinations

Access to the vaccine was initially restricted due to a limited stock of vaccine doses; however, with the import of more doses and the opening of more vaccination sites in community clinics, more people had the opportunity to be immunized. Culturally-adapted interventions have also improved access to vaccines. For example, for specific populations such as Muslims or ultra-orthodox Jews promotion of women-only vaccination sites boosted vaccination rates in females. Another intervention, a mobile vaccine service, has yielded positive results in the AB community, though populations from unrecognized villages have still been consistently neglected. Although these interventions have helped move the campaign forward, logistical difficulties of storage, transport and the rapid vaccine expiration remain obstacles to vaccinating rural minority communities.

### 5.3 Direct Communication With Target Groups

Another important intervention at the beginning of the campaign was directly contacting target groups by phone to urge people to get vaccinated, especially those over 65 years old. While phone calls from volunteers from third sector organizations nudging people to get vaccinated did not produce impressive results, greater success was seen when the calls came from their own general practitioners or community clinics. In a later phase, the intervention turned to workers of the education system.

## 6 Discussion

Since the beginning of the COVID-19 outbreak, multiple aspects of public life have shifted dramatically. Preexisting social inequalities, economic status, and health system imbalances have been exposed and exacerbated by the virus in most countries. Vaccination has been looked to as a beacon of hope to preventing infection and morbidity. However, herd immunity can only be achieved if a large proportion of the population is immunized. In this review, we focus on the challenges and interventions undertaken during the vaccination campaign among a minority population in southern Israel.

Although designing comprehensive plans for mass vaccination are more likely to succeed if culturally adapted. Use of the local language of the minority and involving local councils, community leaders, physicians, and third sector organizations are key to an effective vaccination campaign. Population data collection, vaccination distribution and administration, and general healthcare access should primarily be the responsibility of the health ministry and HMOs. The plan should involve communicating to multiple diverse populations the benefits of vaccination and risks of non-vaccination, and refuting misinformation circulating on social media in a culturally relevant manner. The plan must also tackle the logistics of vaccine availability and methods for delivery to target groups efficiently.

It has been observed that appointing local city or village coordinators charged with community COVID-19 management and vaccination increases vaccination rates. Their advocacy in an effective way for their communities to obtain the resources they need from governing bodies of other parties. Coordinators can also assist with collecting and summarizing relevant local data on the outbreak, coordination and getting all needs and resources from the different parties.

Mobile vaccination clinics have improved vaccine availability, since they operate without the physical constraints of HMOs and their limited work hours. Mobile clinics operating during the evenings or weekends and have been successful in increasing vaccination compliance.

Direct communication with the medical staff, nurses, and physicians also produced successful results, since minority populations tend to be more trusting of their family physicians. Phone calls from general practitioners proved partially successful, though time constraints and intense workloads from acute COVID-19 cases and follow-up made this an unrealistic task for many family physicians.

In addition, health workers can be role models for their communities to increase motivation for vaccination. As previously mentioned, the vaccination rate among medical personnel is high, and this information should be made public in their communities. Taking advantage of the community’s trust in local medical workers, we publicized these data in the hopes of increasing the vaccination rates.

Real-life local data regarding the correlation between vaccination and decline of infections is an additional approach to encouraging vaccination.

Despite the low rate of vaccination among the AB at the beginning of the campaign, the collective effect of these interventions has been an increase in vaccination rates. In addition, higher rates were observed in regions with more detailed plans and intensive interventions. We expected vaccination rates among the AB population to increase gradually until reaching a plateau due to improved awareness and access. A similar pattern was reported during the 2013 polio outbreak in Israel, where vaccination rates climbed up to 98% of eligible Bedouin children in some regions of the Negev as a result of similar interventions [14]. In addition, it might be relevant to know the rate of adherence to treatment of other asymptomatic viral infections in the same population. For example, in a study we conducted, there was no significant difference between the AB and Jewish people living in the Negev regarding adherence to chronic hepatitis B treatment [15].

Moreover, there are factors that impact vaccination rates due to individual decision-making. These factors vary across communities and in different cultural and social contexts. As previously reported, family, religion, health beliefs, socioeconomic status, and ethnicity are all factors that influence adherence to vaccinations [16, 17].

After every crisis, it is paramount that authorities review the results of the implemented interventions to learn from their successes and failures. Reducing healthcare disparities and preparing society for similar potential crises is key to formulating a superior response. Lessons from this outbreak and vaccination campaign can be used to reorder the healthcare system for the benefit of minority populations and establish better preventative practices.

In summary, special considerations are needed to manage routine healthcare for minority populations, but even more so under extraordinary emergent circumstances, such as the need to rapidly vaccinate a large population. The ability to contain future crises requires culturally-adapted awareness campaigns, a mechanism for early identification of the crises, and simulations of an appropriate response. In any region of the world, relevant centralized and local bodies (local councils, appointed coordinators, ministry of health, HMOs, third sector organizations, physicians, and local leaders) should work together when implementing and planning interventions; taking local resources, cultural diversity, and limitations into consideration.

### Conclusion

Cooperation between centralized and local parties is essential for increasing COVID-19 vaccination rates among minority populations. Coordination enables planning, raising awareness, simulations, and execution of interventions to be culturally adapted and tailored to underserved communities. If successful, such coordination will increase vaccine accessibility to minorities and allow interventions to operate more efficiently in future infectious disease outbreaks.
